# Whose emotion is it? Measuring self-other discrimination in romantic relationships during an emotional evaluation paradigm

**DOI:** 10.1371/journal.pone.0204106

**Published:** 2018-09-25

**Authors:** Friedrich Meixner, Cornelia Herbert

**Affiliations:** Applied Emotion and Motivation Psychology, Institute of Psychology and Education, Ulm University, Ulm, Germany; Boston Children's Hospital / Harvard Medical School, UNITED STATES

## Abstract

In healthy subjects, emotional stimuli, positive stimuli in particular, are processed in a facilitated manner as are stimuli related to the self. These preferential processing biases also seem to hold true for self-related positive stimuli when compared to self-related negative or other-related positive stimuli suggesting a self-positivity bias in affective processing. The present study investigates the stability of this self-positivity bias and its possible extension to the emotional other in a sample of *N* = 147 participants including single participants (*n* = 61) and individuals currently in a romantic relationship (*n* = 86) reporting moderate to high levels of passionate love. Participants were presented a series of emotional and neutral words that could be related to the reader’s self (e.g., “my pleasure”, “my fear”), or to an insignificant third person, unknown to the reader (e.g., “his pleasure”, “his fear”) or devoid of any person reference (e.g., “the pleasure”, “the fear”). The task was to read the words silently and to evaluate the word pairs in reference to one’s own feelings elicited during reading. Results showed a self-positivity bias in emotional judgments in all participants, particularly in men. Moreover, participants in a romantic relationship (women and men) evaluated positive, other-related stimuli more often as valence-congruent with one’s own feelings than single participants. Taken together, these findings support the idea of a self-positivity bias in healthy subjects and an expansion of this bias while being in a romantic relationship.

## Introduction

Emotional stimuli are processed in a facilitated manner compared to neutral stimuli. This is a robust finding that could be confirmed in many behavioral and neuroscientific studies for different types of stimuli (e.g., faces, pictures, words; for an overview/review see: [1–3]. Regarding stimulus valence, however, findings often diverge suggesting either preferential (e.g., faster, more accurate or more elaborate) processing of negative stimuli over positive stimuli or vice versa. Regarding the processing of verbal stimuli, many studies support a positivity bias in affective processing in healthy subjects. This bias could be confirmed in neurophysiological studies during passive viewing of positive, negative and neutral words (e.g., [4–6]) and during tasks in which participants were asked to appraise emotional stimuli such as trait adjectives in reference to the self (e.g., [7,8]). Recent research suggests no restriction of this bias to explicit self-evaluation of trait adjectives. Instead, several studies have already demonstrated a self-positivity bias in tasks in which emotional stimuli vary in self-reference: for example, words of emotional and neutral content are presented that are either directly related to the self of the participant or to the self of another person, unknown to the participant (e.g., [9–12]).

Theoretically, self-serving biases [13], mood-congruent processing [14] and a positivity offset (i.e., dominance of approach over avoidance motivation when no imminent threat is present) [15] may facilitate this self-positivity bias. Also, positive mood and absence of imminent threat are being considered the normal experience in most healthy subjects [13,16–20]. In contrast, psychopathological disorders characterized by profound instabilities in mood, affect and self-image such as borderline personality (e.g., [21]), or schizophrenia [22] may diminish the self-positivity bias. Depressive symptoms may even turn the bias into a self-negativity bias [23,24].

Hence, changes in affective state and in self-reference should be strong predictors of whether self-related stimuli are preferentially processed and whether participants prefer positive over negative information. However, little is known about how non-pathological changes in affective experience may influence processing biases of emotional stimuli varying in self-reference. Love, as a biologically grounded, approach- and reward motivated affective state [25–27] may be an ideal testing case to this end.

### Love as a self- and other-oriented affective state

Falling in love can be accompanied by changes in affect and in self-reference. Affective changes may range from intense feelings of passion and euphoria on the one hand, to mania, insecurity and anxiety on the other hand, especially in the initial phase of entering a romantic relationship [25,28]. Therefore, whatever the affective quality experienced in the early-stage of romantic love might be, it may be strongly associated with the ‘emotional other’. For example, according to scientific surveys at least 78–79% of people in love report to think intrusively about the desired person [29]. In fact, when asked to make "me/not me" decisions people in a romantic relationship tend to confuse stimuli related to the self with those related to their loved ones [30]. Consequently, one could assume that falling and feeling in love might also change appraisal of self- and other-related emotional stimuli.

Empirical support for this hypothesis comes from a series of recent experimental studies which found that individuals in love appraise other-related emotional stimuli differently than individuals not in love. For instance, in a series of EEG and behavioral studies by Langeslag and colleagues, individuals in love processed stimuli, e.g., beloved-related vs. friend-related words and phrases in a more elaborate way [31–33]. This intensified processing was observed irrespective of the task. Moreover, with regard to cortical processing (EEG), modulation of late brain potentials such as the LPP, an index of sustained and motivated attention was facilitated in a similar way as would have been expected for preferential processing of self-related and emotional stimuli [30,31]; see also [5]. In line with these findings are results from neuroimaging studies revealing considerable overlap in brain activity during appraisal of information related to the participant’s self or a close other [34]; for related discussion see also [35,36]. The findings suggest diminished self-other discrimination on a neurofunctional level and a decrease of perceived self-other boundaries when thinking of a beloved one. Also, the self-reference effect [37], in general associated with better memory for self-related than other-related stimuli [38], has been found to be significantly reduced when stimuli are appraised in relation to an intimate other (for meta-analysis, see [38]). Moreover, some studies seem to suggest that when in a romantic relationship, personality traits representing the self are confused with personality traits representing the partner [30]. These effects may even extend to other-related stimuli unrelated to the romantic partner [30]. In romantic relationships, this latter effect (extension to other-related stimuli that are unrelated to the partner) might be due to over-representation of the partner when appraising stimuli in the other-related stimulus category. Therefore, it is still unclear whether ‘self-expanding’ effects are partner-specific or whether individuals in love and currently in a romantic relationship are generally more susceptible to incorporating information about other people into one’s own self.

Theoretically, the assumption of self-expansion in individuals in a romantic relationship is well in line with the psychological model of self-expansion by Aron and colleagues (for an overview see [27]). According to this model, love emanates from a basic need of the self to grow and expand by incorporating the other into one’s own mental representation and concept of the self [39]. The expansion of one’s own self when in love is assumed to be an affectively positive experience for many if not all lovers [40,41] facilitating a positivity bias for positive aspects of the partner [25]. This positive bias towards the other is considered to be functional, self-serving and may predict relationship satisfaction [42].

Viewed from an experimental perspective, reading about “his/her fun/joy/success” should therefore elicit similar strong feelings of pleasure in lovers as should reading of self-related positive emotional words. However, as outlined above, it is still unclear if romantic relationships bolster a self-positivity bias while at the same time expanding this bias not only to close and significant others but to others in general. In other words, in individuals in a romantic relationship one would expect a) facilitated processing of other-related emotional words, particularly positive ones–due to the generally increased relevance of other-related information for the lover’s self—plus b) a persisting self-positivity bias in the processing of self-related emotional stimuli. This extension of the self-positivity bias to the emotional other may arise in lovers either due to over-representation of the partner in the other-related stimulus category or because of generally reduced self-other boundaries in lovers, love being a self- and other-oriented positive affective state.

The aim of the present study was to test these assumptions experimentally by investigating changes in response accuracy and reaction times during appraisal of self- and other-related emotional words in *N* = 168 single participants and participants in a romantic relationship and currently in love. We aimed to take a) the relationship status (i.e., being single vs. in a romantic relationship) and, for participants in a relationship, b) feelings of passionate love as well as c) the relationship quality as independent variables into account. This will show, whether a) being in romantic relationship vs. being single will be sufficient to produce an extension of the self-positivity bias and b) whether this effect is dependent on the intensity of love or the quality of the relationship. Experimental stimuli and design were adapted from variants of the His-Mine Paradigm, an experimental paradigm, developed by the corresponding author and shown to robustly measure biases in the processing of self-related and other-related stimuli across tasks and methods including behavioral measures such as response accuracy and reaction times [9,10,21,23,43–45].

In line with the considerations outlined above, the following hypothesis and research questions were tested: (1) Participants in a romantic relationship as well as singles show a self-positivity bias in the appraisal of self-related emotional words, i.e., response accuracy and reaction times to self-related positive words do not differ between the two groups. (2) Compared to singles, participants in a romantic relationship evaluate other-related positive words in a similar way as self-related positive words. This should be reflected in significant group differences in the appraisal of other-related positive words, even in the absence of partner-specific instructions.

## Materials and methods

### Participants

*N* = 168 healthy adults participated in the present study. Participants were recruited via mailing lists and advertisements at the campus of the University of Ulm, Germany. Advertisements contained messages explicitly encouraging individuals currently in a romantic relationship or being single to take part in the study. Participants could take part in the study only if they were heterosexual, 18–30 years of age, neither married nor engaged ever in their lifetime and if they had no children. Participants gave written informed consent and received course credit or 10 euros for participation. The experiment was conducted according to the Declaration of Helsinki and the experimental design was approved by the local ethics committee (https://www.uni-ulm.de/einrichtungen/ethikkommission-der-universitaet-ulm/).

Data from six participants had to be excluded from further analyses because these participants reported acute psychiatric disorders (four participants reported suffering from depression, one from anorexia and one stated suffering from post-traumatic stress disorder). Nine participants had to be excluded because of non-responsive task behavior or technical difficulties and six participants had to be excluded from further analyses because they didn’t indicate their relationship status. Thus, the remaining sample consisted of *N* = 147 healthy adults (94 females, 51 males, 2 unspecified), mean age *M* = 22.33 years, *SD* = 2.70, all native speakers of German.

15 participants indicated they were left-handed, 131 indicated they were right-handed and 1 participant reported being ambidextrous. Right- or left-handedness of participants did not influence reaction times, *t*(144) = .658, *p* = .512, nor average number of valence-congruent responses, *t*(144) = -.048, *p* = .962. Also, right-handedness vs. left-handedness/ambidextrous was not significantly associated with participants’ gender, *Χ*^*2*^ (1) = 3.50, *p* = .061, no expected cell frequencies were below 5. Given that participants were asked to answer with their dominant hand, left-handed and ambidextrous participants remained in the participant sample.

86 participants (24 males) were in a relationship at the time of testing (mean duration *M* = 26.51 months, *SD* = 19.72, range 1–71 months) whereas 61 participants (27 males) indicated they were currently single. Participants in a relationship felt significantly more intense feelings of passionate love for their actual partner on the PLS than singles who had no current partner (*t*(145) = 5.66, *p* ≤ .001). Given that the present study is interested in self-expansion effects elicited during states of being in love with an actual partner as compared to states in which feelings of passionate love are mainly unidirectional (single participants) and at the time of testing unrelated to an actual partner or a relationship, PLS scores were only considered further in participants who were currently in a relationship and felt passionate love for their current partner.

### Materials

The stimulus material consisted of 60 nouns, 20 nouns per emotional valence category (positive, negative and neutral). Nouns were taken from own previous research and matched according to normative ratings of valence and arousal in line with standardized datasets of affective words [46]. In line with previous emotion word processing studies [9,10,23,47] nouns were matched for several linguistic dimensions including word length and word frequency and differed only in valence and arousal, i.e., positive and negative words eliciting higher arousal than neutral nouns. Descriptive statistics of the word material can be found in Table 1. The full list of stimuli is available from the authors upon request.

**Table 1:** Descriptive statistics of the different word categories used in this study.

	Valence	Arousal	Concreteness	Length	Frequency
Positive	7.31 (*0*.*72*) ^a^	4.68 (*0*.*74*) ^a^	4.68 (*0*.*70*) ^a^	5.75 (*1*.*71*) ^a^	263.15 (*283*.*53*) ^a^
Neutral	5.34 (*0*.*67*) ^b^	2.43 (*0*.*97*) ^b^	4.05 (*1*.*83*) ^a^	6.35 (*1*.*35*) ^a^	227.00 (*253*.*77*) ^a^
Negative	2.56 (*0*.*53*) ^c^	4.77 (*0*.*69*) ^a^	4.33 (*0*.*89*) ^a^	6.30 (*1*.*69*) ^a^	206.80 (*189*.*50*) ^a^

^abc^ Different superscripted letters a, b and c indicate statistically significant differences between stimulus categories regarding the respective dimensions (p ≤ .05); mean values are depicted, values in brackets represent standard deviations. Valence, arousal and concreteness range from 1–9 (1: unpleasant/low arousing, abstract/unconcrete; 9: pleasant/high arousing/very concrete). Length represents average number of letters, frequency corresponds to CELEX [48] frequency/per million words).

Each noun of each of the three different valence categories was paired with a possessive pronoun of the first person (“mein/meine”, German word for “my”), a possessive pronoun of the third person (“sein/seine”, German word for “his”) or an article (“der/die/das”, German word for “the”) devoid of any person reference. Although the present sample consisted of male and female participants, who are either single or in a heterosexual relationship, we refrained from using verbal stimuli employing the female pronoun “her” (ihr/ihre) as in German language, “her” (ihr/ihre) is almost indiscernible from “their” (Ihr/Ihre) and may lead to ambiguity. To control if using only male pronouns is influencing how male and female participants, whether single or in love, evaluate emotional stimuli, a manipulation check was included, and gender was taken into consideration in additional analyses (see manipulation check).

**Fig 1:**
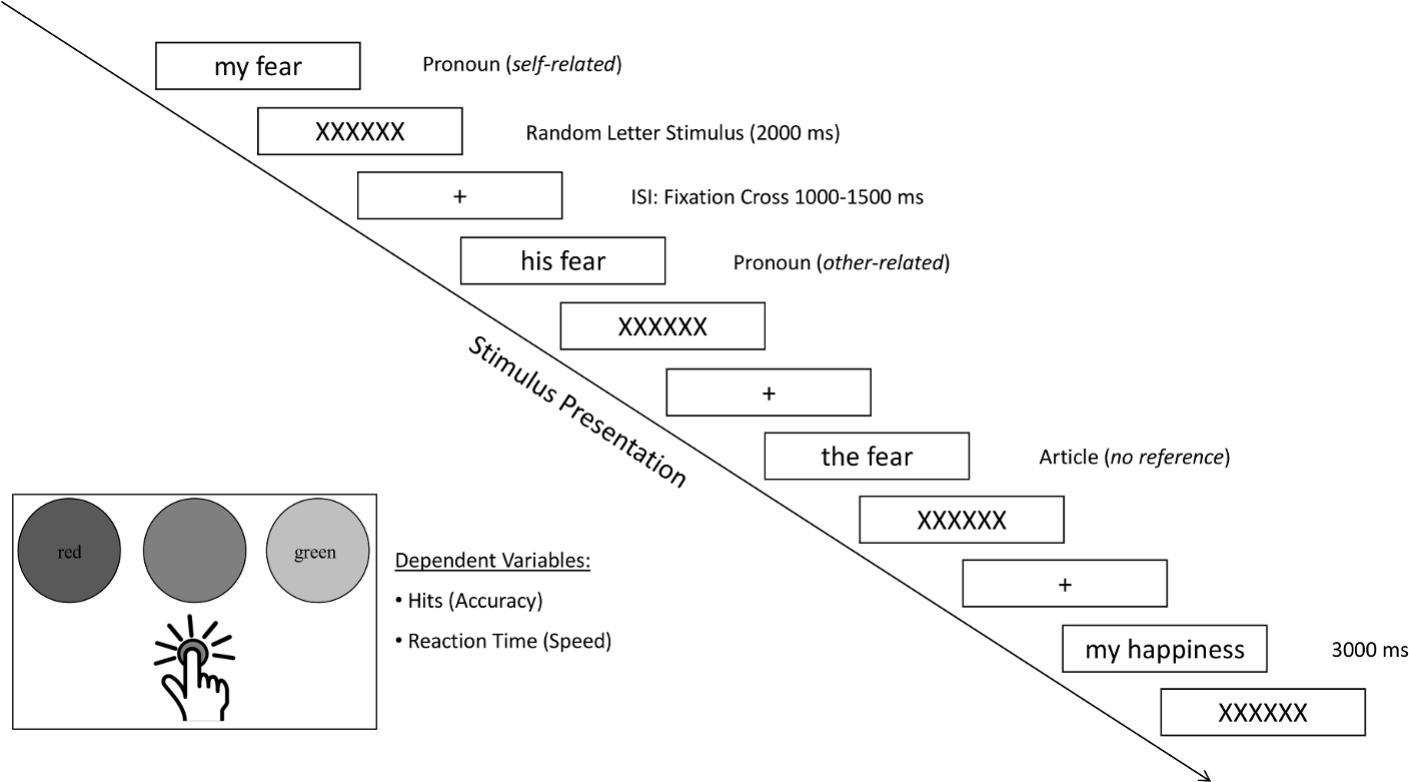
Time course of the experimental paradigm.

Each stimulus pair (pronoun-noun or article-noun pair) was presented in one trial, resulting in a total of 180 trials (see Fig 1 for an overview). Hence, each noun could be related to the reader (e.g., “my fear”, “my joy”, “my furniture” …), to another person, unknown to the reader (e.g., “his fear”, “his joy”, “his furniture” …), or had no personal reference at all (e.g., “the fear”, “the joy”, “the furniture” …), the latter stimulus combinations serving as control stimuli. Each trial started with a fixation cross displayed for a random interval between 1000 and 1500 milliseconds. Word stimuli were presented on a computer screen with a resolution of 1280x1024 pixels at a refresh rate of 60 Hz, in black 70pt letters (Times New Roman) on a white background, with an average viewing distance of 600 mm, resulting in a visual angle of 1° 54' 0.58'' (stimulus height), for 3000 milliseconds or until terminated through the participants pressing one of any answer key. After each word stimulus, a visual stimulus consisting of letter strings (XXXXXX) was presented for 2000 milliseconds to reduce carry over effects from one trial to the other. In addition, given that each noun was presented three times in one run, i.e., either with self-reference, other-reference or devoid of person reference, presentation order of the stimuli was randomized for each participant to avoid sequence effects. Participants had to indicate the valence of the presented stimuli using one of three keyboard buttons. Key presses had to be given with the index finger of the dominant hand, rested on a fixed starting position, equidistant to the three target buttons (Fig 1).

Participants received detailed written and oral instructions. They were told that they would be presented a series of words that could describe emotions or objects belonging to themselves (such as “my fun” or “my furniture”) or to an insignificant third person, unknown to the reader (“…another person, unknown to you”; such as “his fun” or “his furniture”), whereas other words could be personally unrelated (such as in “the fun” or “the furniture”). Regarding other-related stimuli there was deliberately no mention of a romantic partner in the instructions to ensure the measurement of non-partner-specific evaluation of other-related stimuli. Furthermore, participants were instructed to respond spontaneously based on their feelings elicited during reading and—based on these—as accurately and as fast as possible. Before the start of the experiment, participants completed practice trials to ensure they understood the instructions. The whole experimental paradigm lasted about 15–25 minutes. The paradigm was programmed using Presentation® software (Version 0.60, Neurobehavioral Systems, Inc., Berkeley, CA, www.neurobs.com).

### Procedure

Upon arrival at the laboratory, participants provided written informed consent and were questioned about their mental and physical health, they filled in a demographic questionnaire about age, gender, native language, relationship status, and relationship duration. In addition, they answered an anamnestic questionnaire on visual and acoustic impairments, history of neurological/psychiatric disorders. Hereafter, participants completed the experimental paradigm and filled in self-report questionnaires to assess the intensity of feelings of passionate love and the relationship quality in participants in a relationship. These measures included the Passionate Love Scale (PLS; [48–50]), as well as the Relationship Assessment Scale (RAS; [51–53]). The PLS was included to ensure that all participants in the relationship sample were indeed in a *romantic* relationship at the time of testing. The PLS was also filled out by single participants. The PLS is designed such that is it able to measure an individual’s general level of passionate love toward another person (i.e., how passionate you are about someone whom you may be actually in love with (real partner) or whom you were in love with (ex-partner), regardless of whether you’ve actually been in a relationship with that person, or whom someone came closest to caring for in that way).

According to the classifications made by Hatfield & Sprecher [50], *n* = 56 of the participants in a relationship reported to be extremely passionately in love, while *n* = 22 of the participants reported to experience passionate love, whereas only *n* = 8 reported to experience passionate love occasionally and none of the participants scored low or very low on the PLS. The mean score of the PLS (see Table 2) in the romantic relationship sample is comparable to the mean scores reported for exclusively dating couples in Hatfield & Sprecher ([48]; *M*_*men*_ = 215.45, *M*_*women*_ = 220.89). Since all participants reported to experience passionate love at least occasionally, and with the majority of participants in a relationship being passionately in love, it can therefore be safely assumed that all participants in a relationship are experiencing feelings of passionate love for their partner. As shown in Table 1, single participants who were currently not in a romantic relationship scored significantly lower on the PLS than participants in a relationship. Nevertheless, single participants reported moderate scores on the PLS. According to Hatfield & Sprecher’s [50] classification, they are/were also passionate about someone, albeit with less intensity.

**Table 2:** Demographic data and PLS-, RAS-, BDI-II, STAI and empathy scores (all calculated as sum scores as suggested by the respective manuals, except RAS (mean score); reliability coefficients are listed) of the study sample, M (SD) including individuals in a romantic relationship (first line) and those being single (second line).

	Age (*Years*)	Relationship Duration (*Months*)	Passionate Love Scale	Relationship Assessment Scale	BDI-II	STAI (*State*)	STAI (*Trait*)	Empathy
Relationship (*n* = 86)	22.67 *(2*.*78)*^*a*^	26.80 *(19*.*64)*	216.92 *(28*.*24)* ^*a*^	6.08 *(*.*683)*	6.40 *(6*.*55)*^*a*^	33.90 *(7*.*52)*^*a*^	37.21 *(8*.*73)*^*a*^	52.85 *(8*.*06)*^*a*^
Single (*n* = 61)	21.84 *(2*.*50)*^*a*^	-	186.98 (*35*.*74*) ^*b*^	-	6.05 *(5*.*06)*^*a*^	36.77 *(7*.*09)*^*b*^	41.23 *(9*.*59)*^*b*^	52.79 *(7*.*43)*^*a*^
Cronbach’s α	-	-	.938	.907	.842	.851	.890	.789

^*ab*^ Different superscripted letters a and b indicate statistically significant differences between groups regarding the respective dimensions (p ≤ .05); sum scores are depicted, values in brackets represent standard deviations.

In addition, participants filled in the Beck-Depression-Inventory (BDI-II; [54]) as well as the State-Trait-Anxiety Inventory (STAI; [55]) to check for possible subclinical changes in mood and anxiety (see Manipulation Check and Exploratory Analysis). Regarding BDI-scores, data from n = 32 participants were lost due to incomplete data collection. Further control variables included, among others, self-report scales measuring facets of the self-concept [56], and empathy [57,58], including empathic concern to ensure that individuals in a romantic relationship did not differ significantly in these measures from singles. A detailed overview of the demographic and individual characteristics of the study sample, including reliability coefficients (Cronbach’s α) for the scales of interest is provided in Table 2.

### Analysis: Response accuracy and reaction times

Participants’ evaluative judgments including response accuracy (i.e., number of valence-congruent responses) and reaction times were statistically analyzed with repeated measures analysis of variance (ANOVA) using IBM SPSS Statistics 24 software and a 3 x 3 x 2 full factorial design. The full factorial design included the factors *stimulus valence* (positive, negative, neutral) and *stimulus reference* (self, other, no personal reference) as within-subject factors and *relationship status* (single, relationship) as between-subject factor, differentiating individuals currently in a romantic relationship and in passionate love with their partner from individuals being single. For reaction times, latencies were kept in their raw units (milliseconds) and only valence-congruent trials were included in the calculation of mean reaction times. Different sample sizes (and consequently different degrees of freedom) in analyses of reaction times compared to analyses of accuracy are due to some participants having never evaluated a certain stimulus category in a valence-congruent way (e.g., having never evaluated neutral stimuli such as “my shoes” with a “neutral” button press), therefore having no reaction times for these stimuli, which excludes them from the ANOVAs using reaction times as the dependent value. However, since valence-incongruent answers are still valid answers even if zero valence-congruent responses were obtained in a certain stimulus category, this was no exclusion criterion.

Degrees of freedom were corrected using the Greenhouse-Geisser correction, if sphericity was violated. Significant within-subject effects in the ANOVA designs were further analyzed using paired samples t-tests, significant between-subject effects were analyzed using independent samples t-tests. If homogeneity of variances was not given, Welch’s t-test was calculated and reported instead. All reported p-values are uncorrected. If multiple comparisons were performed apart from analyses testing the main hypotheses, Bonferroni correction was applied. Measures of effect size (Partial Eta Square, ƞp^2^) and 95% confidence intervals are reported for the ANOVAs and post-hoc t-tests and for the manipulation check, but not in the exploratory analyses.

### Manipulation check

Given our heterosexual sample and that only the male category was used in the other-related stimulus category, additional ANOVAs (including *gender* as a between-subject factor, together with the factor *relationship status*) were performed with response accuracy (number of valence-congruent responses) and reaction times as dependent variables. Moreover, participants were asked open questions about whom they were thinking when judging self- and other-related stimuli to assess the extent of intrusive thinking of the partner, particularly when appraising stimuli in the other-related stimulus category.

### Exploratory analysis

To determine whether effects may be mediated by interindividual differences in control variables (e.g., relationship satisfaction, empathic concern, mood states including depressive symptoms and anxiety), mediation analyses and Pearson correlation coefficient analyses were performed (two-sided testing with *p* ≤ 0.05 as significance criteria) and Bonferroni correction was applied.

## Results and discussion

Since every stimulus category consisted of 20 pronoun-noun pairs, a maximum number of 20 valence-congruent responses was possible. Mean accuracy of the sample was *M* = 15.37 (*SD* = 2.48), which equals 76.85% valence-congruent responses across all categories. Mean reaction time was *M* = 1284.44 ms (*SD* = 317.35) across all categories.

### Positivity bias–self–other: Effects of relationship status

#### Response accuracy (number of valence-congruent responses)

The 3 (stimulus valence) x 3 (stimulus reference) x 2 (relationship status) repeated measures ANOVA (dependent value, DV: response accuracy) revealed significant main effects of the factors *stimulus valence* (*F*(1.66, 241) = 111.37, *p* ≤ .001, ƞp^2^ = .434), and *stimulus reference* (*F*(1.74, 252) = 28.05, *p* ≤ .001, ƞp^2^ = .162), as well as significant interactions between *stimulus valence* and *relationship status* (*F*(1.66, 241) = 3.37, *p* = .045, ƞp^2^ = .023), and between *stimulus valence* and *stimulus reference* (*F*(2.73, 395) = 36.76; *p* ≤ .001, ƞp^2^ = .202), as well as between *stimulus valence* and *stimulus reference* and *relationship status* (*F*(2.73, 395) = 4.67, *p* ≤ .001, ƞp^2^ = .048).

Post-hoc tests of the interaction between *stimulus valence* and *stimulus reference* revealed a self-positivity bias in line with our hypothesis (1): Within-subject comparisons revealed that positive stimuli were associated with significantly more valence-congruent responses when they were self-related as compared to when they were other-related or presented without any person reference (all participants: self-positive (*M* = 17.63, *SD* = 3.20) vs. other-positive (*M* = 15.62, *SD* = 4.72); *t*(146) = 6.11; *p* ≤ .001; self-positive vs. unreferenced-positive (*M* = 16.80, *SD* = 3.44); *t*(146) = 4.90; *p* ≤ .001). Also, positive, self-related stimuli were responded to more accurately compared to neutral, self-related stimuli but accuracy (number of valence-congruent responses) did not differ between positive, self-related and negative, self-related words (all participants: self-positive vs. self-negative (*M* = 17.80, *SD* = 4.07); *t*(146) = .49, *n*.*s*.; self-positive vs. self-neutral (*M* = 9.97, *SD* = 4.87); *t*(146) = 14.16, *p* ≤ .001). As can be seen in Table 3, this pattern does not differ significantly for single participants and participants in a romantic relationship, indicating that both groups demonstrate a strong self-positivity bias.

**Table 3:** Valence-congruent answers: Paired samples t-test results for differences between selected stimulus categories for singles and participants in a romantic relationship.

					95% CI	
Pair	*M*	*SD*	*t*		Lower Bound	Upper Bound
Single Participants^a^						
self-positive—other-positive	3.39	4.20	6.30	***	2.32	4.47
self-positive—self-negative	-0.08	3.90	-0.16		-1.08	0.92
self-positive—self-neutral	6.84	7.58	7.04	***	4.89	8.78
self-positive—unreferenced-positive	0.80	1.70	3.69	***	0.37	1.24
Participants in a Romantic Relationship^b^						
self-positive—other-positive	1.02	3.53	2.69	**	0.27	1.78
self-positive—self-negative	-0.23	4.39	-0.49		-1.17	0.71
self-positive—self-neutral	8.24	5.70	13.41	***	7.02	9.47
self-positive—unreferenced-positive	0.84	2.25	3.45	***	0.35	1.32

^a^df = 60

^b^df = 85

**p ≤ .01

***p ≤ .001

**Fig 2:**
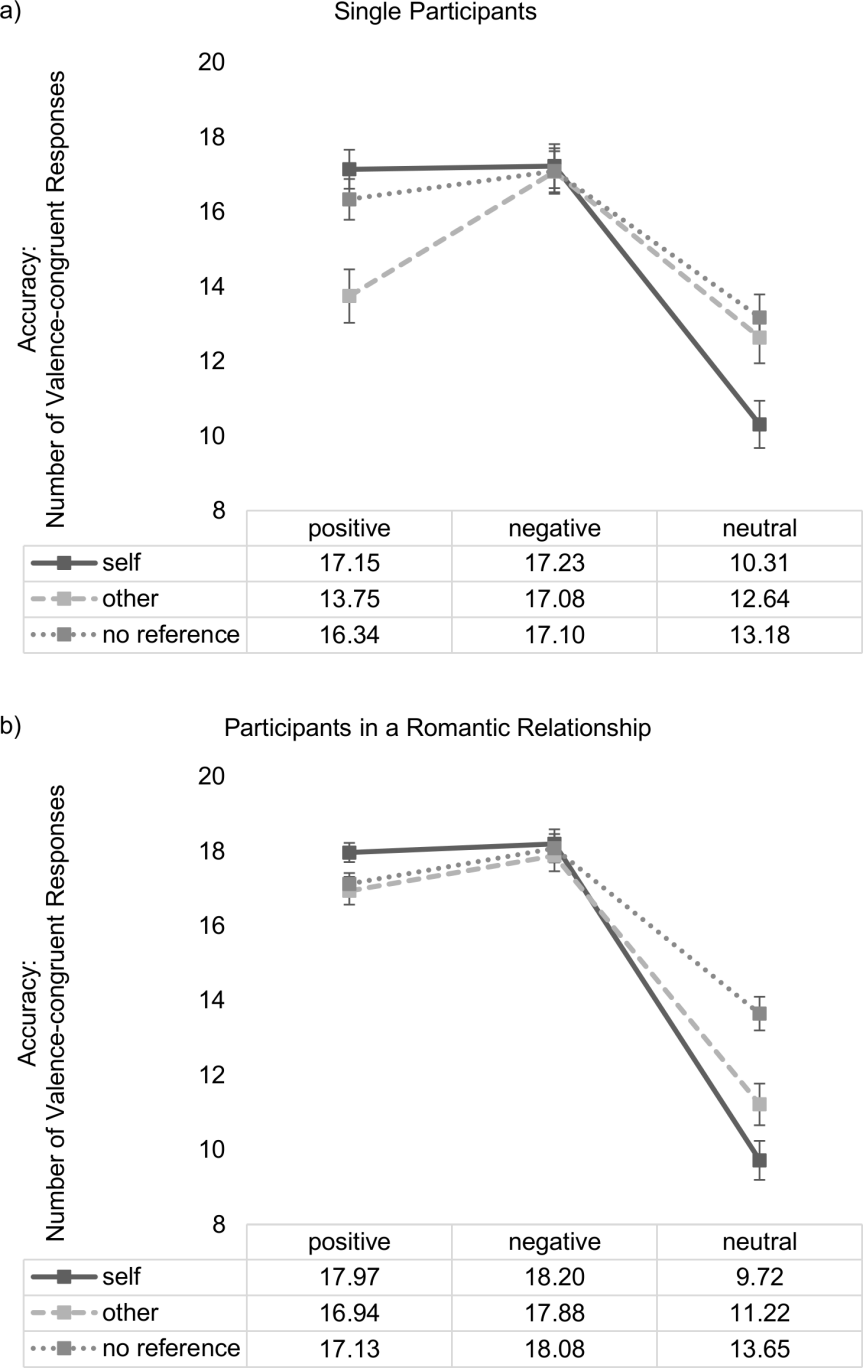
**Stimulus valence x stimulus reference x relationship status interaction (DV: accuracy) for singles (a) and participants in a romantic relationship (b).** Vertical bars denote +/- standard errors.

**Fig 3:**
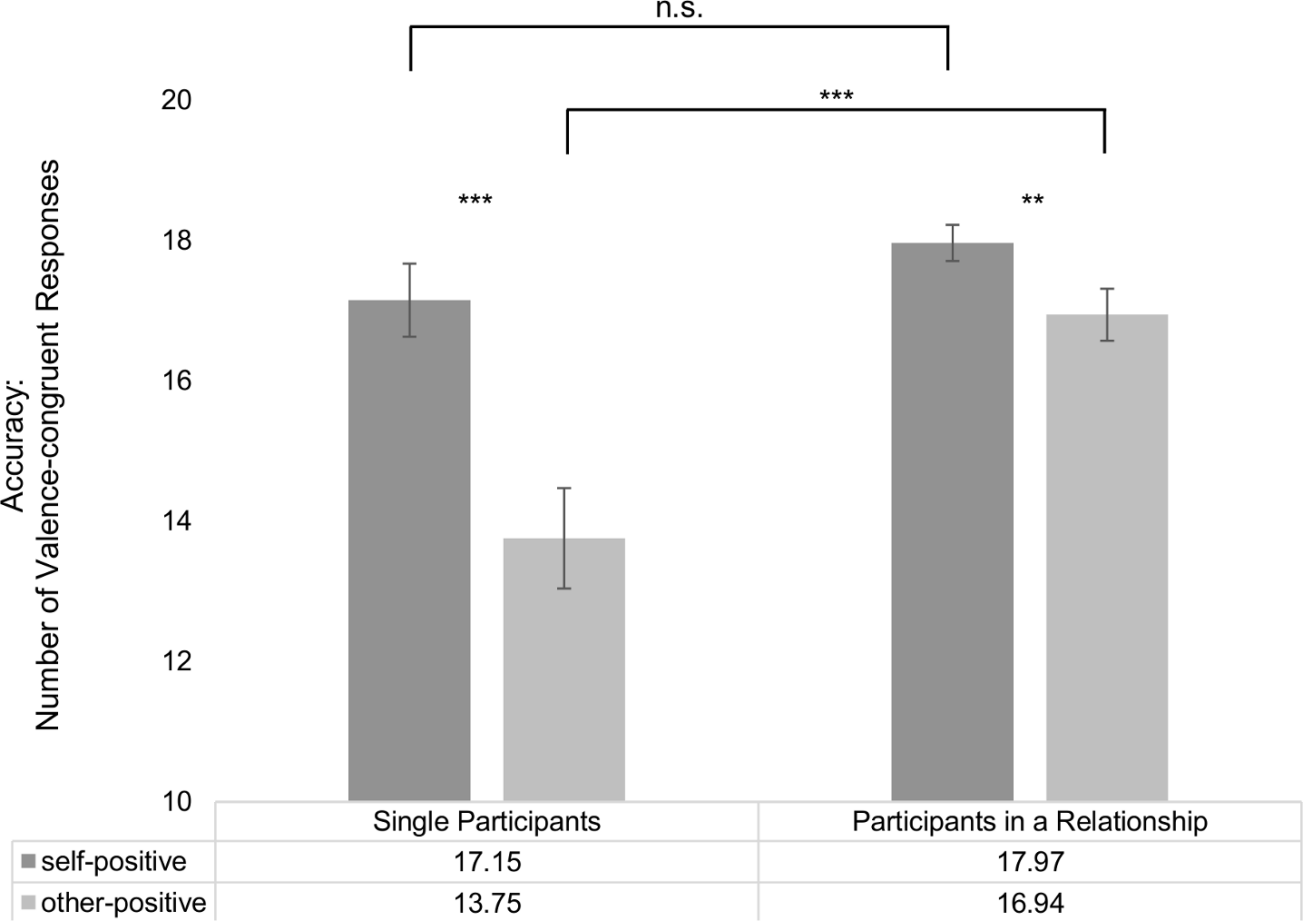
Accuracy for positive words with self-reference vs. other-reference; group comparison: Control group of singles compared to participants in a romantic relationship. Vertical bars denote +/- standard errors.

However, between-subject comparisons revealed that participants in a romantic relationship were significantly more accurate in the categorization of positive, other-related stimuli than were single participants (participants in a romantic relationship: *M* = 16.94, *SD* = 3.44; single participants: *M* = 13.75, *SD* = 5.59; *t*(91.93) = 3.96, *p* ≤ .001) whereas both groups showed no significant differences in the evaluation of positive, self-related stimuli (participants in a romantic relationship: *M* = 17.97, *SD* = 2.39; single participants: *M* = 17.15, *SD* = 4.06; *t*(89.31) = 1.41, *p* = .16). Effects are summarized in Table 3, Fig 2 and Fig 3. Consequently, regarding accuracy data, the difference score between “positive, other-related stimuli” and “positive, self-related stimuli” also displayed a significant group difference between single participants and participants in a romantic relationship (participants in a romantic relationship: *M* = -1.02, *SD* = 3.53; single participants: *M* = -3.39, *SD* = 4.20; *t*(114) = 3.60, *p* ≤ .001).

#### Reaction times

**Fig 4:**
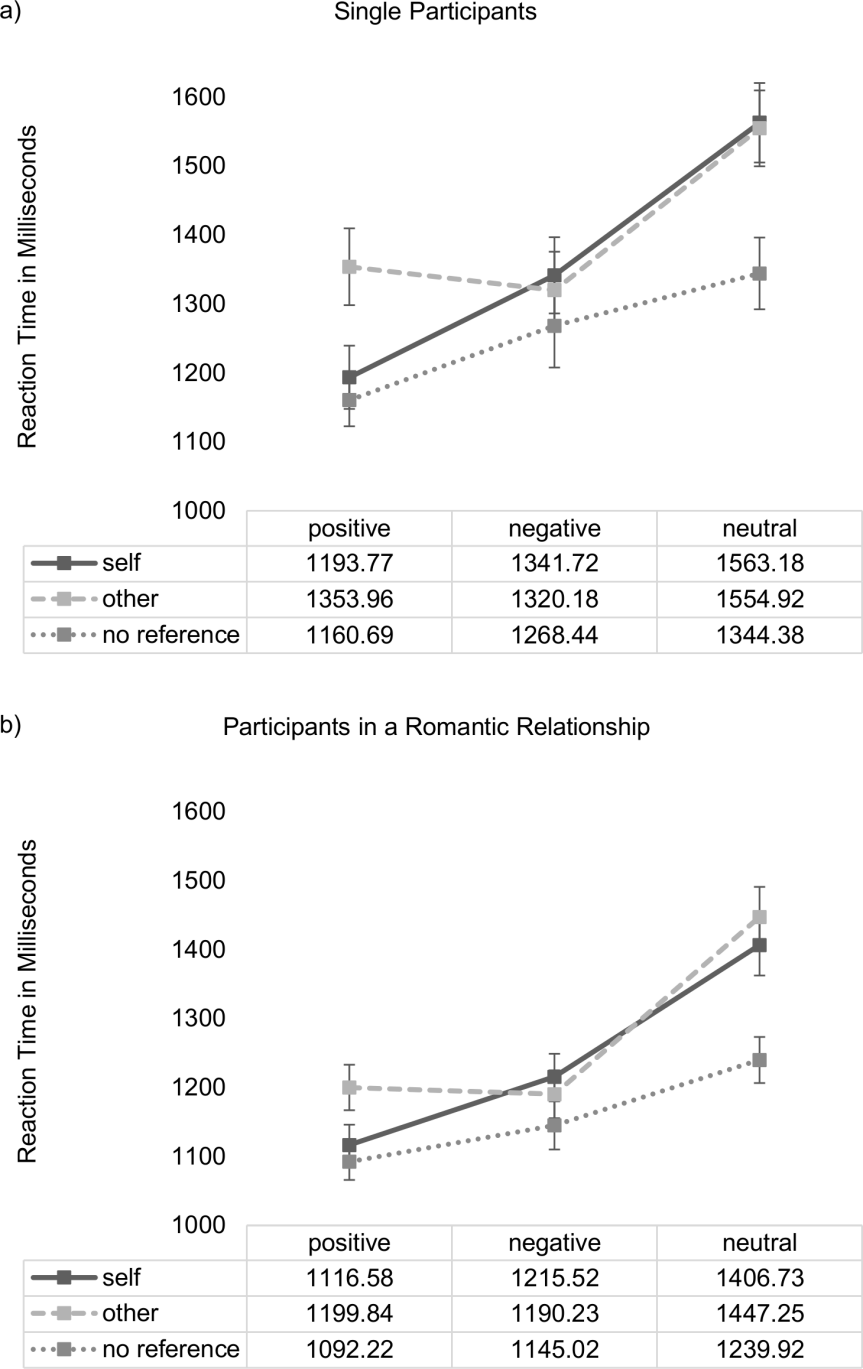
**Stimulus valence x stimulus reference x relationship status interaction (DV: mean reaction time in milliseconds) for singles (a) and participants in a romantic relationship (b).** Vertical bars denote +/- standard errors.

Analysis of reaction times revealed significant main effects of *stimulus valence* (*F*(1.81, 245) = 77.32, *p* ≤ .001, ƞp^2^ = .364), *stimulus reference* (*F*(1.98, 267) = 71.51, *p* ≤ .001, ƞp^2^ = .346), and *relationship status* (*F*(1,135) = 5.02, *p* = .027, ƞp^2^ = .036), as well as significant interaction effects between *stimulus valence* and *stimulus reference* (*F*(3.18, 430) = 17.34; *p* ≤ .001, ƞp^2^ = .114) but, as shown in Fig 4, no significant three-way interaction between *stimulus valence*, *stimulus reference* and *relationship status* (*F*(3.18, 430) = 1.53; *p* = .191, ƞp^2^ = .011). Using log-transformed data instead of raw data did not influence the direction, magnitude or significance of these findings.

Post-hoc-tests of the interaction between *stimulus valence* and *stimulus reference* revealed that reaction times for self-related positive stimuli were significantly faster than were reaction times for self-related negative or self-related neutral words or reaction times for other-related positive words (all participants: self-positive (*M* = 1146.59, *SD =* 295.45) vs. self-negative (*M =* 1276.30, *SD =* 355.17); *t*(143) = 6.61, *p* ≤ .001; self-positive (*M =* 1152.17, *SD =* 298.01) vs. self-neutral (*M =* 1501.14, *SD =* 540.40); *t*(142) = 9.46, *p* ≤ .001; self-positive (*M =* 1146.94, *SD =* 295.37) vs. other-positive (*M =* 1252.11, *SD =* 353.73); *t*(142) = 5.62, *p* ≤ .001). As shown in Fig 4 these effects did not differ significantly between groups (single participants vs. participants in a romantic relationship).

### Manipulation check and exploratory analysis

#### Gender effects

To check for possible gender effects, an additional 3 (*stimulus valence*) x 3 (*stimulus reference*) x 2 (*gender*) ANOVA (DV: response accuracy) was performed. The ANOVA revealed significant main effects of the factors *stimulus valence* (*F*(1.62, 231) = 97.61, *p* ≤ .001, ƞp^2^ = .406), and *stimulus reference* (*F*(1.74, 250) = 24.09, *p* ≤ .001, ƞp^2^ = .144), as well as significant interactions between *stimulus valence* and *gender* (*F*(1.61, 231) = 10.69, *p* = .045, ƞp^2^ = .070), and between *stimulus valence* and *stimulus reference* (*F*(2.83, 405) = 39.40; *p* ≤ .001, ƞp^2^ = .216), as well as between *stimulus valence*, *stimulus reference* and *gender* (*F*(2.83, 405) = 13.94, *p* ≤ .001, ƞp^2^ = .089). Between-group comparisons between women and men demonstrated a significant difference in the number of valence-congruent answers to positive, other-related words (male participants: *M* = 14.25, *SD =* 5.63; female participants: *M =* 16.37, *SD =* 4.03; *t*(143) = -2.62, *p* = .010). Women showed higher accuracy (number of valence-congruent responses) for positive, other-related words than men. Within-subject comparisons (positive self-related vs. positive other-related words; positive self-related vs. neutral self-related words) showed a self-positivity bias in both male and female participants (see S1 Table and S1 Fig).

Regarding reaction times, the 3 (*stimulus valence*) x 3 (*stimulus reference*) x 2 (*gender*) ANOVA revealed significant main effects of *stimulus valence* (*F*(1.80, 239) = 71.42, *p* ≤ .001, ƞp^2^ = .349), *stimulus reference* (*F*(1.98, 263) = 72.88, *p* ≤ .001, ƞp^2^ = .354), *gender* (*F*(1,133) = 11.42, *p* ≤ .001, ƞp^2^ = .079), and significant interaction effects between *stimulus valence* and *stimulus reference* (*F*(3.20, 425) = 14.15; *p* ≤ .001, ƞp^2^ = .096). Post-hoc tests suggest that women responded faster than men overall, regardless of the stimulus category (male participants: *M* = 1374.07, *SD =* 341.24; female participants: *M =* 1230.06, *SD =* 293.85; *t*(143) = 1.83, *p* = .009). Effects are depicted in S2 Fig.

Interactions between relationship status and gender were tested in a 3 (*stimulus valence*) x 3 (*stimulus reference*) x 2 (*relationship status*) x 2 (*gender*)-ANOVA (DV: response accuracy), which showed main effects of *stimulus valence* (*F*(2, 282) = 95.13, *p* ≤ .001, ƞp^2^ = .403) and *stimulus reference* (*F*(2, 282) = 24.80, *p* ≤ .001, ƞp^2^ = .150) as well as interaction effects of *stimulus valence* and *stimulus reference* (*F*(4, 564) = 40.70, *p* ≤ .001, ƞp^2^ = .224), *stimulus valence* and *gender* (*F*(2, 282) = 9.92, *p* ≤ .001, ƞp^2^ = .066), *stimulus valence* x *stimulus reference* x *gender* (*F*(4, 564) = 11.29, *p* ≤ .001, ƞp^2^ = .074), *stimulus valence* x *stimulus reference* x *relationship status* (*F*(4, 564) = 4.43, *p* ≤ .01, ƞp^2^ = .030), but no significant interaction of the factors *gender* and *relationship status* (*F*(1, 141) = 2.02, *p* = .158, ƞp^2^ = .014) and no significant four-way interaction (*F*(4, 564) = 1.05, *p* = .382, ƞp^2^ = .007).

Analyzing reaction times in a 3 (*stimulus valence*) x 3 (*stimulus reference*) x 2 (*relationship status*) x 2 (*gender*)-ANOVA, revealed significant main effects of *stimulus valence* (*F*(2, 262) = 68.59, *p* ≤ .001, ƞp^2^ = .344), *stimulus reference* (*F*(2, 262) = 70.19, *p* ≤ .001, ƞp^2^ = .349), *relationship status* (*F*(1, 131) = 4.27, *p* ≤ .05, ƞp^2^ = .032) and *gender* (*F*(1, 131) = 9.78, *p* ≤ .01, ƞp^2^ = .069). Furthermore, there were interaction effects between *stimulus valence* and *stimulus reference* (*F*(4, 524) = 13.80, *p* ≤ .001, ƞp^2^ = .095), but neither a significant interaction between *stimulus valence*, *stimulus reference* and *relationship status* (*F*(4, 524) = 1.93, *p* = .105, ƞp^2^ = .014), *stimulus valence*, *stimulus reference* and *gender* (*F*(4, 524) = 1.42, *p* = .227, ƞp^2^ = .011), nor between factors *gender* and *relationship status* (*F*(1, 141) = 2.02, *p* = .510, ƞp^2^ = .003), nor between *stimulus valence*, *stimulus reference*, *relationship status* and *gender* (*F*(4, 524) = 1.75, *p* = .138, ƞp^2^ = .013).

#### Manipulation check

Post-experimental questions asking whom participants had imagined while judging other-related stimuli showed that *n* = 62 (55 female, 7 male participants) reported having thought at least occasionally about their partner, ex-partner or friends and family members while evaluating other-related stimuli. Notably, this occurred in participants in love as well as in single participants.

#### Exploratory analysis

**Positivity Bias–Self–Other: Effects of Passionate Love:** To assess whether feelings of passionate love influence the positivity bias to other-related positive stimuli and how this might interact with participants’ relationship status (singles vs. being in a romantic relationship) correlation analyses were performed for the whole sample as well as separately for each group (singles vs. participants in a romantic relationship). Regarding participants in a romantic relationship, correlation analyses also included relationship quality (RAS scores). Correlation analyses were performed for accuracy in positive, other-related stimuli. Group-specific analyses revealed that neither PLS scores (single participants: *r* = .165, *p* = .202; participants in a romantic relationship: *r* = -.040, *p* = .718) nor RAS scores (*r* = -.017, *p* = .895) were correlated with the accuracy in other-related positive stimuli. Across all participants, however, PLS was positively correlated with the accuracy in positive, other-related stimuli (*r* = .212, *p* = .010).

A mediation analysis was conducted to investigate whether passionate love mediates the effect of relationship status on the accuracy in positive, other-related stimuli. Results indicated that relationship status (single participants vs. participants in a romantic relationship) was a significant predictor of PLS score, *b* = 29.94, *SE* = 5.28, *p* < .001, and that the PLS score was a significant predictor of the accuracy in positive, other-related stimuli, *b* = .029, *SE* = .011 *p* = .010. However, relationship status was still a significant predictor after controlling for participants’ PLS score, *b* = 2.28, *SE* = .825, *p* < .001. About 12% of the variance in accuracy in positive, other-related stimuli could be explained by the predictors (*R*^2^ = .118). The indirect effect was tested using a bootstrap estimation (5000 samples), which indicated that the indirect effect was not significant (*b* = .342, *SE* = .375; 95% *CI* = -.388, 1.12).

Moreover, in this non-clinical sample, there were no significant correlations between relationship duration, relationship satisfaction, empathic concern, depressive symptoms or state/trait anxiety and overall reaction times (all *p* > .05). Also, correlation analyses of reaction times taking *stimulus valence* and *stimulus reference* into account showed no significant correlations with the aforementioned variables as well (all *p* > .05).

Regarding accuracy (valence-congruent responses), significant negative correlations were found between overall number of valence-congruent responses and measures of trait anxiety (*r* = -.217, *p* = .008) and depressive symptoms (*r* = -.300, *p* = .003), but there were no significant correlations with relationship duration, relationship satisfaction, empathic concern, nor state anxiety. Regarding depression, results could be obtained only for a subset of the participants for why results have to be taken with caution.

Analyzing the relations between control variables revealed that intensity of love (PLS) was positively correlated with relationship satisfaction (*r* = .425, *p* ≤ .001), and relationship duration (log-transformed; *r* = .317, *p* ≤ .01), but not with empathic concern (*r* = .11, *p* = .175), state anxiety (*r* = -.10, *p* = .288), nor with trait anxiety (*r* = -.03, *p* = .715) or depressive symptoms (*r* = .085, *p* = .369). Differences between single participants and participants in romantic relationship are reported in Table 2. All p-values reported in this section are reported uncorrected and significant effects also held true after Bonferroni correction.

## Discussion

The present study investigated how a romantic relationship and being passionately in love with a partner influences the appraisal of self- and other-related emotional stimuli. Following previous research and taking Aron et al.’s theoretical model of self-expansion [30] into account it was investigated whether participants in a romantic relationship will show an extended self-positivity bias towards other-related, positive stimuli even if these stimuli are related to third persons in general. In addition, it was investigated whether appraisal of self-related, positive stimuli and hence, the self-positivity bias would be unaffected by this and it was explored how feelings of passionate love as well as gender and relationship duration affect the appraisal of self- and other-related emotional stimuli.

The presented results suggest that participants in a relationship showed intense feelings of passionate love towards their partner. Their PLS scores were comparable to scores of exclusively dating couples reported in Hatfield & Sprecher [48]. PLS scores of singles were moderate in height indicating that singles may experience feelings of passionate love to some degree but in an unreciprocated fashion and not with the same intensity as participants in a romantic relationship. Regarding appraisal of self-related words, results revealed a self-positivity bias. This bias occurred in both participant samples (singles vs. individuals in a romantic relationship), which was evident in response accuracy (number of valence-congruent responses) and reaction times. Comparisons within each group (see Figs 2 and 3 and Table 3) revealed that singles as well as individuals in a romantic relationship showed significantly more valence-congruent judgments for positive words when these were self-related as compared to when these were other-related or presented without any person reference. Moreover, singles as well as individuals in a romantic relationship responded significantly faster to self-related positive words than to self-related negative or neutral words. This suggests that a processing bias towards positive, self-related content exists independently from being in a romantic relationship. Moreover, this positivity bias for self-related positive words cannot be explained by confounding linguistic dimensions as the same set of nouns was presented in each reference condition; additionally, words were carefully matched on several linguistic dimensions previously shown to affect emotional word processing.

However, between-subject comparisons (i.e., when individuals in a romantic relationship were compared to singles) revealed an extended self-positivity bias, specifically for other-related positive words in individuals in a romantic relationship compared to singles. This bias was evident in response accuracy: as shown in Fig 3 individuals in a romantic relationship showed more valence-congruent judgments for positive, other-related words than singles. This was observed in the absence of instructions hinting specifically at a close other (i.e., romantic partner). Thus, the extended positivity bias from self-related to other-related words in individuals in a romantic relationship may point towards an increased relevance of other-related, positive stimuli in general, irrespective of the personal significance of the ‘other’. This would be in accord with the hypothesis of overall reduced self-other boundaries regarding positive emotions when in a romantic relationship. Interestingly, individuals in a romantic relationship were generally faster in their emotional evaluations than singles. This did, however, not interact with better accuracy for other-related, positive words in individuals in a romantic relationship compared to singles: the main effect did not interact with *stimulus valence* or *stimulus reference*, and neither could a three-way interaction between *stimulus valence*, *stimulus reference* and *relationship status* be found.

Intrusive thinking about the partner in individuals in a romantic relationship could explain the observed group differences in the processing of other-related positive words. When questioned post-experimentally, 55 participants in total, reported having thought occasionally about their partner, ex-partner, friends or close others when appraising stimuli in the insignificant-other-related stimulus categories. Intrusive thinking about the partner or about any other close relative cannot fully explain why specifically individuals in a romantic relationship appraised other-related positive words more often in a valence-congruent fashion, compared to singles. However, future studies are needed to validate this finding. To this end, an additional experimental condition should be included which explicitly instructs participants to think about their partners or ex-partners. This might help to further examine reduced self-other boundaries in individuals in a romantic relationship regarding their specificity towards the romantic partner compared to generalized effects towards an unspecified other.

Also, gender might play a role: exploratory analysis taking participants’ gender as an additional factor into account revealed overall faster reaction times for significant interactions between the factors *stimulus valence* and *gender*, as well as an interaction between *stimulus valence*, *stimulus reference* and *gender*. Notably, there was no significant interaction between *stimulus reference* and *gender*, neither for response accuracy nor for reaction times. This might be interpreted as evidence that women and men did not respond differently to the masculine form of the pronouns used in this paradigm. Women compared to male participants, however, show an increased accuracy in the evaluation of specifically positive, other-related words compared to male participants while not differing in their evaluation of positive, self-related words. Therefore, male participants showed greater differences in the evaluation of positive, self-related words compared to other-related positive words. This suggests a stronger self-positivity bias in men compared to women. Regarding the specificity of the reported self-expansion effects in romantic relationships, it can be assumed that gender might have a similar facilitating effect on the general accuracy in the evaluation of other-related positive stimuli. Yet, no significant interactions containing both *gender* and *relationship status* were found. Therefore, although gender may bias the interaction between the emotionality of a word and its reference during emotional evaluation, the expansion of the self-positivity bias in participants in a romantic relationship was unrelated to any possible effects caused by the participants’ gender.

As hypothesized, it appears that in the present study, the self-positivity bias is expanded towards other-related words when individuals in a romantic relationship are compared to single participants. This seems to occur, even in the absence of instructions directing thoughts towards close others (i.e., romantic partner) and is unrelated to the participants’ gender, although the latter might have a similar effect. Being in love may expand the processing bias for self-related stimuli towards other-related stimuli. This self-other overlap would be in line with the *self-expansion theory* by Aron et al. [27,30,59]. Moreover, the present results suggest that this effect might not be limited towards the close other. Instead, this effect may rather lead to a broadening of the processing bias towards positive, other-related stimuli in general.

### Limitations and future directions

Since the present study was quasi-experimental in nature, the results should be interpreted accordingly. While we did find significant group differences in the responses towards other-related positive stimuli comparing single participants and participants in a romantic relationship, this obviously does not imply causation. Participants in a relationship might differ from singles in traits other than those measured and that we had controlled for in the present study. For instance, it is likely that singles and participants in a relationship differ in traits that influence why some people seek for and stay in a romantic relationship. It could be, that such traits may also influence the evaluation of self- and other-related information processing. A longitudinal study investigating participants in love over the course of forming, maintaining and maybe also terminating a romantic relationship while measuring changes in self-reported romantic love, relationship satisfaction and experimental reactions towards self- and other-related stimuli could provide insight into the mechanisms underlying love-related changes in the appraisal of self- and other-related stimuli. Moreover, extending participant samples to non-Western cultural samples might be especially fruitful to determine cultural differences in love-related changes in self-other evaluation.

On a related note, the presented results are a first step in understanding the exact mechanics underlying the extension of the self-positivity bias in romantic relationships. While it could be demonstrated that relationship status alone does influence the emotional evaluation of positive, other-related stimuli, and that neither relationship duration nor relationship quality mediate this effect, the exact mechanism and exact role of passionate love in this process still need to be uncovered. Although no correlation of intensity of passionate love and valence-congruent appraisal of other-related, positive words occurred, this might simply be because the present participants included in the relationship sample were homogenously passionately in love. It might be interesting to replicate the present experimental study with a broader sample of participants, covering all of Hatfield & Sprecher’s [50] proposed categories of intensity of passionate love as well as participants in long-term romantic relationships over decades of their lives. Interestingly, the present sample of young, unmarried participants with academic backgrounds in relatively short-term relationships displayed a positive correlation between relationship duration and PLS scores. The intensity of their passionate feelings might be the reason for these couples to stay together in the first place. In long-term relationships, however, there might be a decline in certain aspects of passionate love [60] over time, reflecting a change in the nature of the relationship, which points towards the possibility that appraisal of self- and other-related stimuli might also change after years or decades of living in a romantic relationship.

In future studies, one may also include singles and participants in a relationship who are matched in their intensity of passionate love, which is reciprocated in one case and not in the other. This would allow for a proper evaluation of any effects of passionate love on the degree of enhancement in the processing of positive, other-related stimuli. However, participants in a relationship who aren’t at least somehow passionately in love might terminate their romantic relationship sooner or later, making them rare to find. Furthermore, experimentally manipulating or assessing experienced closeness would allow for an elaborate distinction between effects of passionate love and effects of mere closeness with another person such as a friend, a relative, or, oppositely, a stranger. Additionally, real-world scenarios are often much more complex than solely appraising certain stimuli such as words in the laboratory. Moreover, besides passionate love, other variables such as context, attitude towards the “other” and recent interpersonal interactions might influence emotional evaluation in real life.

## Conclusion

As outlined in detail above, being in a romantic relationship extends the self-positivity bias to positive other-related stimuli. This expanded self-positivity bias, now a self-other-positivity bias, supports the self-expansion model proposed by Aron and colleagues [27,30,61]. The observed behavioral differences cannot be alternatively explained by differences in relationship duration or relationship satisfaction, or by interindividual differences in empathic concern, depressive symptoms or anxiety. In summary, our results suggest that positive emotions regarding another person might increase in relevance if a person is in love with another person and shares a relationship with him/her. Hence, the theoretically described effects of self-expansion and broadening of self-reference to another person when in love [38] seem to be emotion-specific, particularly to positive emotions and not limited to memory effects [38]. Taken together, the present results add to the notion that also non-pathological changes in affective experience can influence processing biases of emotional stimuli varying in self-reference.

## Supporting information

S1 Fig **Stimulus valence x stimulus reference x relationship status interaction (DV: accuracy) for male (a) and female participants (b).** Vertical bars denote +/- standard errors.
(PDF)

S2 Fig **Stimulus valence x stimulus reference x relationship status interaction (DV: mean reaction time in milliseconds) for male (a) and female participants (b).** Vertical bars denote +/- standard errors.
(PDF)

S1 Table Valence-congruent answers: Paired samples t-test results for differences between selected stimulus categories for male and female participants. (PDF)
