# Supplementary figures and images for: Whose emotion is it? Measuring self-other discrimination in romantic relationships during an emotional evaluation paradigm
Source: PLoS One. 2018 Sep 25;13(9):e0204106. doi: 10.1371/journal.pone.0204106 (PMC6155531; doi:10.1371/journal.pone.0204106)

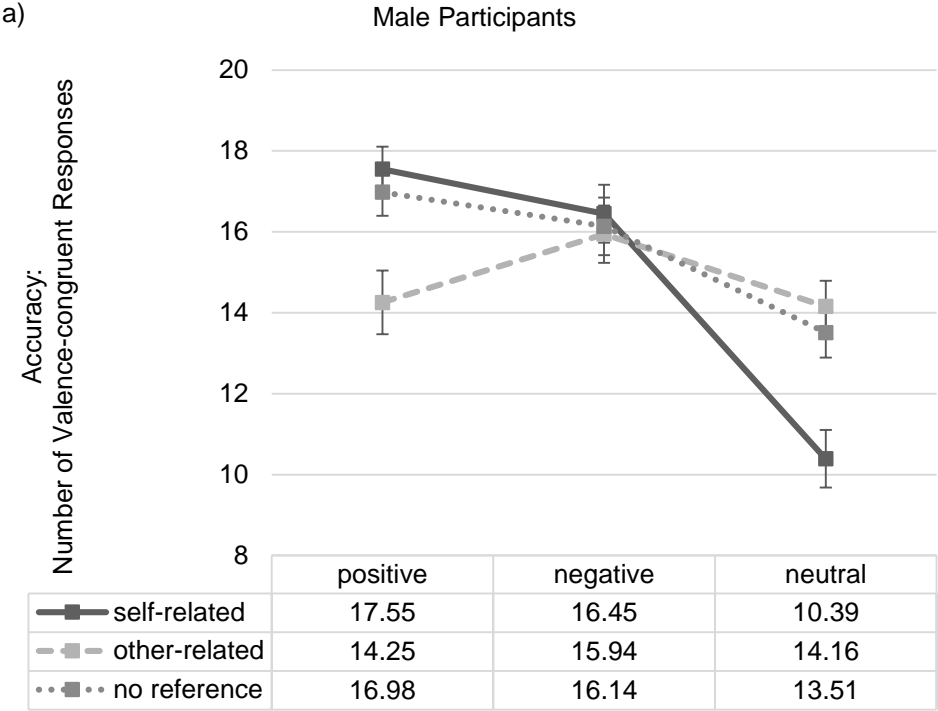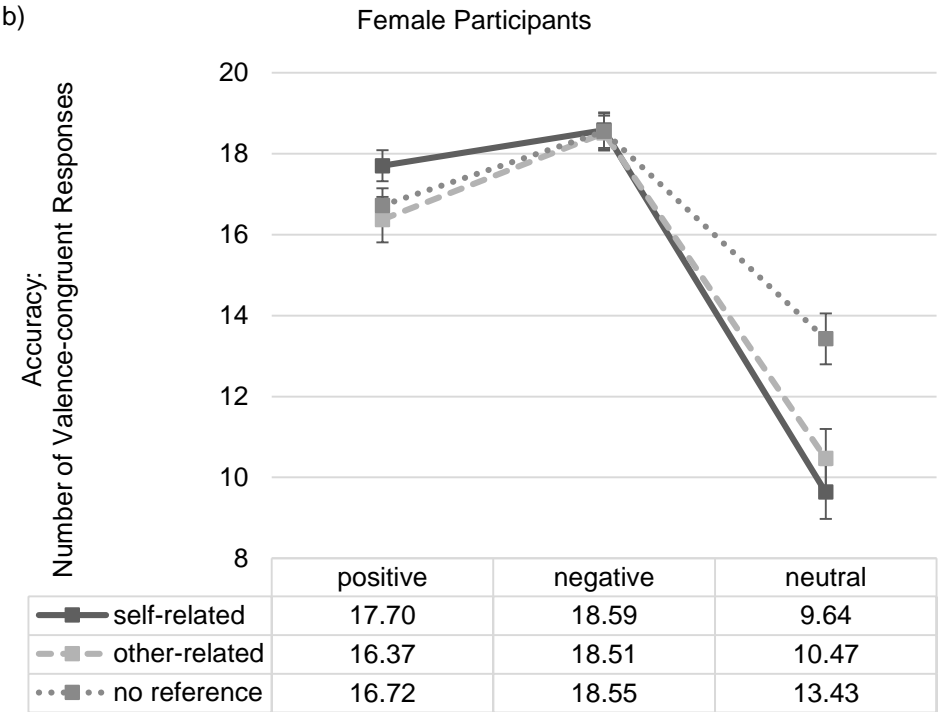

Supplement: S1 Fig — Stimulus valence x stimulus reference x relationship status interaction (DV: accuracy) for male (a) and female participants (b). Vertical bars denote +/- standard errors. (PDF) [file pone.0204106.s001.pdf]

a)

## Male Participants

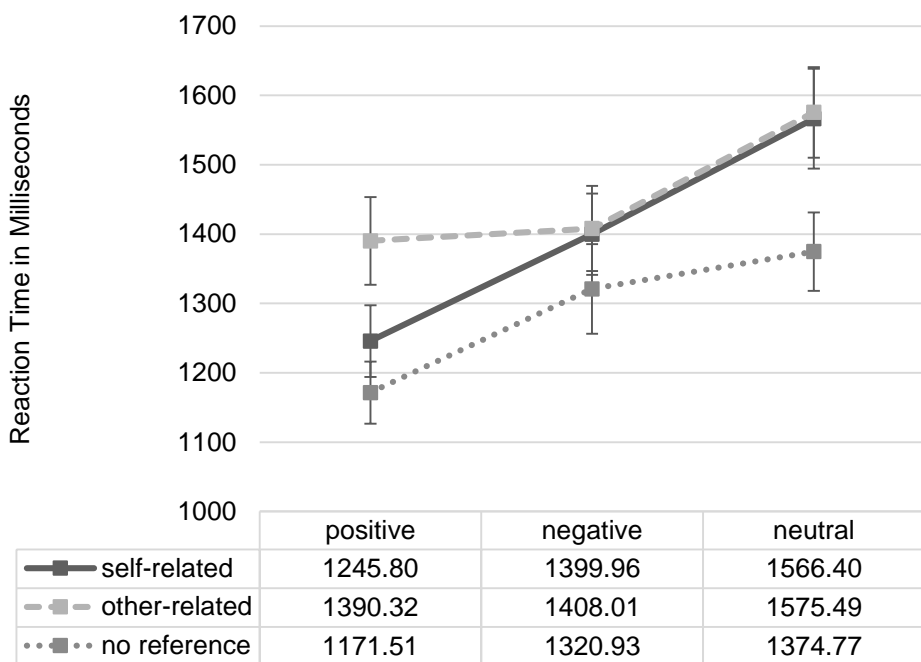

b)

## Female Participants

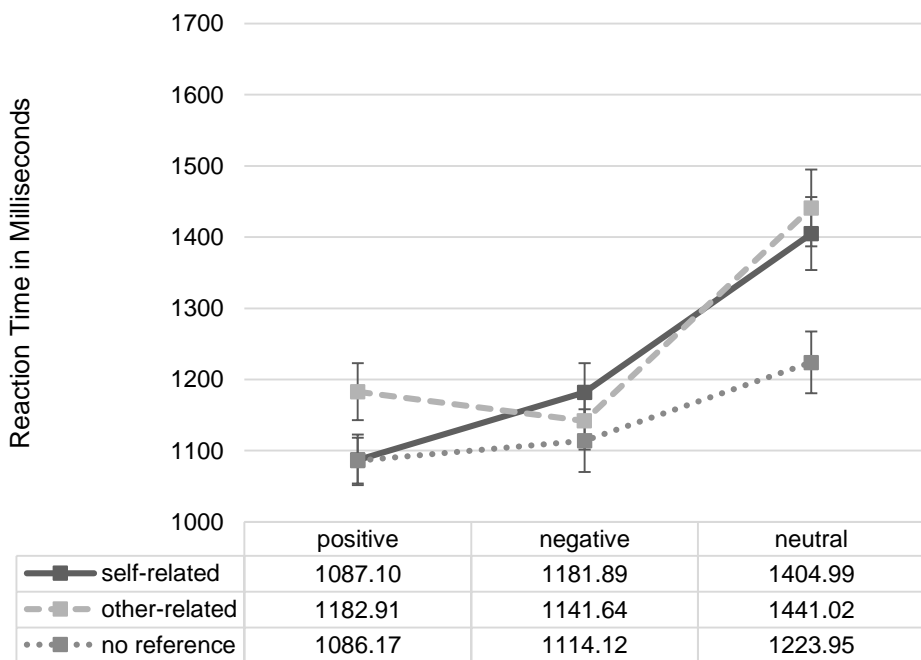

Supplement: S2 Fig — Stimulus valence x stimulus reference x relationship status interaction (DV: mean reaction time in milliseconds) for male (a) and female participants (b). Vertical bars denote +/- standard errors. (PDF) [file pone.0204106.s002.pdf]
