# Supplementary material for: Whose emotion is it? Measuring self-other discrimination in romantic relationships during an emotional evaluation paradigm
Source: PLoS One. 2018 Sep 25;13(9):e0204106. doi: 10.1371/journal.pone.0204106 (PMC6155531; doi:10.1371/journal.pone.0204106)
Supplement: S1 Table — (PDF) [file pone.0204106.s003.pdf]

S1 Table. Valence-Congruent Answers: Paired Samples t-Test Results for Differences between Selected Stimulus Categories for Male and Female Participants.

					95% CI	
Pair	*M*	*SD*	*t*		Lower Bound	Upper Bound
Male Participants[a]						
self-positive - other-positive	3.29	4.72	4.98	***	1.97	4.62
self-positive - self-negative	1.10	3.78	2.07	*	0.03	2.16
self-positive - self-neutral	7.16	7.25	7.05	***	5.12	9.19
self-positive - unreferenced-positive	0.57	1.90	2.14	*	0.03	1.10
Female Participants[b]						
self-positive - other-positive	1.33	3.40	3.80	***	0.63	2.03
self-positive - self-negative	-0.88	4.24	-2.02	*	-1.75	-0.01
self-positive - self-neutral	8.06	6.17	12.67	***	6.80	9.33
self-positive - unreferenced-positive	0.98	2.11	4.49	***	0.55	1.41

[a]df = 50
[b]df = 93
*$p \leq .05$
**$p \leq .01$
***$p \leq .001$
